# Detection of Viral Hemorrhagic Septicemia Virus (VHSV) from *Diporeia *spp. (Pontoporeiidae, Amphipoda) in the Laurentian Great Lakes, USA

**DOI:** 10.1186/1756-3305-4-2

**Published:** 2011-01-06

**Authors:** Mohamed Faisal, Andrew D Winters

**Affiliations:** 1 Department of Fisheries and Wildlife, Michigan State University, S-112 Plant Biology Building, East Lansing, MI, 48824 USA; 2 Department of Pathobiology and Diagnostic Investigation, Michigan State University, 4125 Beaumont Road, Lansing, MI, 48910 USA

## Abstract

The mode of viral hemorrhagic septicemia virus (VHSV) transmission in the Great Lakes basin is largely unknown. In order to assess the potential role of macroinvertebrates in VHSV transmission, *Diporeia *spp., a group of amphipods that are preyed upon by a number of susceptible Great Lakes fishes, were collected from seven locations in four of the Great Lakes and analyzed for the presence of VHSV. It was demonstrated that VHSV is present in some *Diporeia *spp. samples collected from lakes Ontario, Huron, and Michigan, but not from Lake Superior. Phylogenetic comparison of partial nucleoprotein (N) gene sequences (737 base pairs) of the five isolates to sequences of 13 other VHSV strains showed the clustering of *Diporeia *spp. isolates with the VHSV genotype IVb. This study reports the first incidence of a fish-pathogenic rhabdovirus being isolated from *Diporeia*, or any other crustacean and underscores the role macroinvertebrates may play in VHSV ecology.

## Findings

The viral hemorrhagic septicemia virus (VHSV), genotype IVb, is a recent invader to the Laurentian Great Lakes basin and has been associated with mortalities in a number of resident freshwater fish species [1], [2], [3], [4]. While laboratory studies demonstrated that the virus can be transmitted to naïve fish by both immersion and injection [5], [6], [7], the mode of VHSV transmission in the Great Lakes basin is largely unknown. In a previous study, it was concluded the pisocolid intermittent leech *Myzobdella lugubris *harbors VHSV [8]. Whether other macroinvertebrates can act as a vector or reservoir for VHSV remains to be elucidated.

In the Great Lakes foodweb, amphipods of the genera *Diporeia*, *Gammarus*, and *Hyalella *occupy a central position as they transform energy from lower to higher trophic levels [9]. Unfortunately, *Diporeia *spp. have experienced a sharp decline sharp decline in abundance over the last two decades [10]; the cause(s) of which puzzle scientists. To tackle this enigma, a study was designed that involved comprehensive parasitological and microbiological analysis of *Diporeia *spp. collected from lakes Ontario, Huron, Michigan and Superior [Faisal and Winters: Pathogens impacting *Diporeia *spp. in the Great Lakes, submitted].

**Figure 1:**
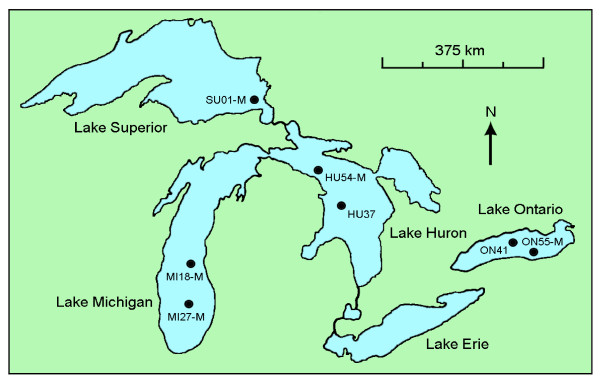
**Map of the Laurentian Great Lakes showing where *Diporeia *spp. were collected for this study**. The solid circles denote sampling locations.

*Diporeia *spp. were collected between August 2007 and April 2008 by taking Ponar grabs from seven locations in the Great Lakes basin at depths between 74-190 meters. The approximate locations of collections of *Diporeia *spp. are shown in Figure 1. Collected *Diporeia *spp. were pooled (five amphipods/pool), immersed briefly in absolute ethanol for surface disinfection, and then rinsed several times in sterile water. Samples (~100 μg) were homogenized with a sterile mortar and pestle and then diluted with 1 ml Earle's salt-based minimal essential medium (MEM, INVITROGEN). Homogenized *Diporeia *contents were removed with a sterile transfer pipette, dispensed into a sterile 1.5 ml centrifuge tube, and centrifuged at 5500 rcf for 20 min and supernatants were immediately used for virus isolation.

Since there is currently no amphipod cell line that could be used to aid in the isolation of amphipod-pathogenic viruses, virus isolation was performed according to the standard protocols detailed in the American Fisheries Society Blue Book [11] and the Office International des Epizooties [12], using the *Epithelioma papulosum cyprinii *(EPC) cell line [13]. Inoculated 96-well plates containing EPC cells grown with MEM (5% fetal bovine serum) were incubated at 15°C for 21 days, and were observed for the formation of cytopathic effects (CPE). Second and third blind passages were performed and assessed for the presence of CPE. All cell culture positive samples of *Diporeia *homogenates (ON41, ON55-M, HU54-M, MI18-M, and MI27-M) caused CPE on EPC in the form of focal areas of rounded, refractile cells which progressed to full lysis of the cell monolayer.

Reverse transcriptase polymerase chain reaction (RT-PCR) was then performed on all samples (Table 1) Total RNA was extracted from inoculated cell culture supernatant using a QIAamp^® ^Viral RNA Mini Kit (QIAGEN). Reverse transcription was accomplished by a two-step protocol using the Affinity Script Multiple Temperature Reverse Transcriptase RT-PCR™ (AGILENT TECHNOLOGIES). The primer set used in this assay was recommended by the Office de International Epizootics for the detection of a 811 base pair sequence of the VHSV nucleocapsid (N) gene: 5'-GGG GAC CCC AGA CTG T-3' (forward primer) and 5'-TCT CTG TCA CCT TGA TCC-3' (reverse primer). Amplicons of 811 base pairs were amplified in all cell culture positive samples.

**Table 1:** Locations in the Laurentian Great Lakes from which Diporeia spp. were collected for this study (CPE = formation of cytopathic effect; RT-PCR = results for amplification of the viral hemorrhagic septicemia virus nucleoprotein gene).

Lake	Station	Depth (m)	Latitude	Longitude	Date	CPE	RT-PCR
Ontario	ON41	129	43°43.0000 N	078°01.6299 W	4/25/08	+	+
	ON55-M	190	43°26.6000 N	077°26.2903 W	4/25/08	+	+
Huron	HU37	74	44°45.7001 N	082°47.0103 W	8/7/07	-	-
	HU54-M	124	45°31.0000 N	083°25.0301 W	8/7/07	+	+
Michigan	MI18-M	160	42°44.0601 N	086°59.9803 W	4/16/08	+	+
	MI27-M	103	43°36.0101 N	086°55.0002 W	4/18/08	+	+
Superior	SU01-M	95	46°59.5601 N	085°09.6300 W	8/18/07	-	-

**Figure 2:**
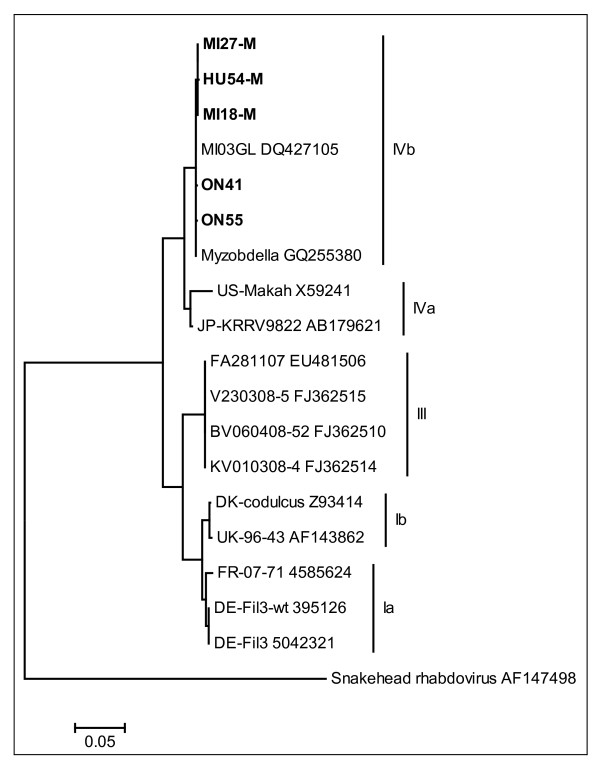
**Distance tree constructed for phylogenetic comparison of isolates obtained in this study**. The tree generated using the Neighbor-joining algorithm and maximum likelihood method shows high phylogenetic similarity between the five viral hemorrhagic septicemia virus (VHSV) isolates obtained in this study (ON41, ON55-M, HU54-M, MI18-M, and MI27-M) and other isolates belonging to the VHSV genotype IVb. The alignment file used to produce the tree contained partial VHSV nucleoprotein (N) gene sequences (737 nucleotide positions). Snakehead rhabdovirus was used as the outgroup. The scale bar indicates the number of substitutions per nucleotide site.

The RT-PCR yielded five samples (ON41, ON55-M, HU54-M, MI18-M, and MI27-M) with the characteristic VHSV 811 bp band. The amplicons were further purified with the Wizard^® ^SV Gel and PCR Clean-up System (PROMEGA) and then sequenced from both directions. Overlapping sequences for each isolate were aligned using the BioEdit contig assembly program version 7.0.9.0 [14] and the aligned contigs were used for multiple alignments performed by ClustalW [15]. Phylogenetic analysis of the VHSV *Diporeia *strain with 14 nucleoprotein encoding genes from other species of rhabdovirus was done by generating the phylogenetic dendrogram (Figure 2) using MEGA 4 [16] and the Neighbor-Joining algorithm [17]. Phylogenetic analysis of the five *Diporeia *isolate sequences (737 bp) base pairs [GenBank: HQ214133-HQ214135, HQ415762-HQ415763] showed that the sequences clustered with the VHSV IVb-MI03 strain, the index strain of the Great Lakes VHSV [GenBank: DQ427105]. In comparison to the Great Lakes VHSV strain, two nucleotide substitutions were observed in *Diporeia *isolates from both lakes Huron and Michigan: a transition from cytosine to thymine at nucleotide position 408 which caused a silent mutation and a transversion from guanine to thymine at nucleotide position 907 which caused a mutation from glycine to cysteine.

Our findings provided evidence that VHSV can exist within *Diporeia *spp. Whether VHSV propagates in the cells of these amphipods or just existed in their viscera or gills is currently unknown and deserves further investigation. Indeed, the presence of VHSV in *Diporeia *spp. in three of the four Great Lakes sampled is surprising since these amphipods were collected from depths that ranged from 74-190 meters where none of the susceptible fish are known to reside. *Diporeia *spp. feed on detritus and planktonic organisms and is known to scavenge for food items in lower depths. Such a feeding habit has the potential to transfer VHSV from the benthos to the pelagic zone through their excreta or by being preyed upon by susceptible fish; lake whitefish for example. Moreover, based exclusively on data generated in this study, one cannot rule out that VHSV is a pathogen of *Diporeia *spp. or that *Diporeia *spp. can be a reservoir for VHSV in the Great Lakes. Regardless of these currently unanswered questions, this study reports the first incidence of a fish-pathogenic rhabdovirus being isolated from *Diporeia*, or other crustacean. This finding underscores the dire need to better understand the role of macroinvertebrates in disease ecology.

## Competing interests

The authors declare that they have no competing interests.

## Authors' contributions

AW conducted field collection. AW and MF, designed the study, performed all laboratory assays, and drafted the manuscript. Both authors reviewed and approved of the final manuscript.
